# Assessment of three types of surgical procedures for supravalvar aortic stenosis: A systematic review and meta-analysis

**DOI:** 10.3389/fcvm.2022.987522

**Published:** 2022-09-06

**Authors:** Lizhi Lv, Xinyue Lang, Simeng Zhang, Cheng Wang, Qiang Wang

**Affiliations:** ^1^Center for Pediatric Cardiac Surgery, Fuwai Hospital, National Center for Cardiovascular Diseases, Chinese Academy of Medical Sciences and Peking Union Medical College, Beijing, China; ^2^Medical Research and Biometrics Center, National Center for Cardiovascular Diseases, Fu Wai Hospital, Chinese Academy of Medical Sciences and Peking Union Medical College, Beijing, China

**Keywords:** single-patch repair, two-patch repair, three-patch repair, supravalvar aortic stenosis, network meta-analysis

## Abstract

**Importance:**

The safety and efficacy of different surgical repairs of supravalvar aortic stenosis (SVAS) are inconsistent.

**Objective:**

To compare the prognosis of single-, two- and three-patch repair for patients with SVAS.

**Data sources:**

PubMed, EMBASE, Cochrane Library, Web of Science, and clinicaltrials.gov were searched until April 17, 2022.

**Study selection:**

Study reported SVAS patients treated with single-, two- or three-patch repair.

**Data extraction and synthesis:**

Two reviewers independently extracted the data of study characteristics and clinical outcomes. Multiple pairwise and frequentist network meta-analyses were conducted. And a fixed-effect model was used when no heterogeneity existed.

**Main outcomes and measures:**

Outcomes included the rate of reintervention, aortic insufficiency, early mortality and late mortality, cardiopulmonary bypass (CPB) time, cross-clamping (CCP) time, and postoperative/ follow-up pressure gradient. Binary variables were evaluated by odds ratio (OR) and its 95% confidence interval (CI), while continuous variables were assessed by standardized mean difference (SMD) and its 95% CI.

**Results:**

Twenty-seven retrospective cohort studies were included, comprising 1,162 patients, undergoing single-patch (46.6% of cases), two-patch (33.9%), and three-patch repair (19.4%). Two-patch method had a lower rate of reintervention compared with single-patch (OR = 0.47, 95 % CI 0.28–0.89), and three-patch (OR = 0.31, 95 % CI 0.15–0.64). This finding also applied to juvenile and non-Asian patients. Three-patch method had a lower rate of aortic insufficiency compared with single-patch (OR = 0.11, 95 % CI 0.01–0.63), and two-patch (OR = 0.11, 95 % CI 0.02–0.83). But this repair had the longest CCP time, which was significantly longer than that of single- (SMD = 0.76, 95 % CI 0.36–1.17) or two-patch repair (SMD = 0.61, 95 % CI 0.06–1.16). No significant difference was found in mortality and pressure gradient among three procedures.

**Conclusion and relevance:**

Two-patch repair has the lowest reintervention rate and relatively reasonable operation time. Complex and severe SVAS is suggested to be treated with two-patch repair. Further prospective studies of a reasonable sample size will be required with a special focus on the use of different patch materials and surgeons' unique working experience.

**Systematic review registration:**

http://www.crd.york.ac.uk/PROSPERO/, identifier: CRD42022328146.

## Introduction

Congenital supravalvular aortic stenosis (SVAS) is a rare obstructive lesion of the left ventricular outflow tract (LVOT), the pathogenesis of which may be related to Williams Burren syndrome (WBS) (1). Morphologically, SVAS is characterized by the presence of stenosis of the sinotubular junction (STJ). Diagnostically, SVAS is classified as type I (discrete) or type II (diffuse) (2) depending on the degree of aortic arch involvement, with the former manifesting as limited hourglass-like stenosis at the STJ (3), while the latter includes more uniform stenosis of the STJ and ascending aorta, and may involve with the aortic arch or descending aorta. Greutmann et al. (4) considered that adult patients with SVAS usually require intervention because of aortic valve problems, although the progression of their stenosis is relatively rare. It is generally accepted that juvenile patients with SVAS would need to be treated as soon as possible because of the progressive nature of their stenosis (5–9).

The majority of patients with SVAS require surgical interventions, but their procedures may vary case by case. There are three main categories of aortic root correction procedures: the single-patch repair (McGoon repair), the two-patch repair (Doty repair), and the three-patch repair (Brom repair). The single patch repair was a teardrop-shaped patch used for the aortic root augmentation after longitudinal incision through the stenotic site extending to non-coronary sinus (10). The two-patch repair involved a pantaloon-shaped patch plasty (11). The three-patch repair enlarged the aortic root into three aortic sinuses with three separate “Shield”-shaped patches (12). To date, however, there is no expert consensus regarding the safety and efficacy of different procedures for the surgical correction of SVAS. In the evolution of surgical procedures for SVAS, single-patch repair predominated early on, followed by two-patch repair, and currently, three-patch repair was the mainstay (13). However, authors also found that the dominant treatment modality in Europe and the United States is now two-patch and three-patch repair (14–18), whereas single-patch and two-patch repairs are predominant in Asian countries (19–22). Different surgeons have their preferred surgical approaches (13). Accordingly, this review systematically summarized and assessed the similarities and differences as well as relative superiority of three different types of surgical corrections for SVAS in clinical settings to keep surgeons updated on the substantial evidence for the surgical correction of SVAS.

## Methods

### Strategy for literature retrieval

The meta-analysis was carried out under the guidance of the Preferred Reporting Items for Systematic reviews and Meta-Analyses (PRISMA) statement (23, 24) (PROSPERO ID CRD42022328146). A total of five English language databases—PubMed, EMBASE, Cochrane Library, Web of Science, and ClinicalTrials.gov—were searched to identify all potentially eligible studies up to April 17, 2022. The search terms were (“single-patch” or “two-patch” or “three-patch”) and “supravalvar aortic stenosis” (The detailed search strategy was available in the Supplementary material).

### Literature selection

Abstracts of the included citations were screened by two reviewers (L-Z.L. and X=Y.L.) using Endnote (v9.3.1) and if appropriate, full-length articles were downloaded to identify the potentially eligible studies. The discrepancies were resolved by an additional senior reviewer S-M.Z. If multiple publications from the same cohort were available, we included the publication with the longest follow-up period. The detailed inclusion and exclusion criteria of the articles were as follows: a case-control study or cohort study, diagnosed as SVAS, treated with single-patch repair, two-patch repair, or three-patch repair, reported the outcomes of each of the patch-repair methods, at least 3 cases reported per repair method, and full texts available. The following studies were excluded when they were conference abstracts, case reports, review articles, or animal studies.

### Data extraction and quality assessment

Baseline information and outcome data of the eligible studies were collected including first author name, year of publication, country, study design (cohort or case-control association study), surgery method, the sample size in each operation group, patients age at operation, the percentage of female, type I SVAS, WBS, and pulmonary artery stenosis (PAS), pre-operative, post-operative and follow-up transvalvular pressure gradient, follow-up time, the rate of lost follow-up, intraoperative cardiopulmonary bypass (CPB) time and cross-clamping (CCP) time, the number of reintervention, postoperative aortic insufficiency, early mortality (in-hospital/within 30 days), and late mortality. Two reviewers extracted the data independently and compared the results to ensure coherence. The discrepancies were resolved by the third reviewer S-M.Z.

Methodological Index for Non-Randomized Studies (MINORS) (25) was carried out by two reviewers (L-Z.L. and X-Y.L.) to independently assess the quality of the non-randomized studies. The MINORS tool represents a 12-item assessment system of the methodological value, with 8 criteria indicated for non-comparative studies and 4 criteria indicated for comparative studies. Each criterion was scored from 0 to 2. The items were scored 0 (not reported), 1 (reported but inadequate), or 2 (reported and adequate). The global ideal score was 16 for non-comparative and 24 for comparative studies.

### Statistics analysis

The outcomes were the events of reintervention, post-operative aortic insufficiency, early mortality, late mortality, post-operative and follow-up transvalvular pressure gradient, intraoperative CPB time, and CCP time. We performed multiple pairwise meta-analyses (direct effect) and frequentist network meta-analysis (26) to assess the effectiveness of single-, two-, and three-patch repair treatment. Binary variables were evaluated by odds ratio (OR) and its 95% confidence interval (CI), while continuous variables were evaluated by standardized mean difference (SMD) and its 95% CI. If the original data appearing in the literature was represented by the median and interquartile range (IQR), then the median was approximately represented by the mean, the IQR was converted into the standard deviation (SD) value, and the calculation formula was SD = IQR/1.35. The 95% CIs were used for both OR and SMD values. If the number of events for both sets of comparisons was zero, we added 0.05 to the value to conduct the process of merging according to Haldane correction (27). The *I*-square, Tau-square, Cochran Q statistic, and its corresponding *p*-value were used to test the heterogeneity. And if the *I*-square was >50% or the *p*-value of Tau-square and Cochran Q statistic was <0.05 (28), the heterogeneity among the studies was considered and the random-effects model was used for analysis, otherwise the fixed effects model was used. The linear regression method (Egger test) (29) and funnel plots were used to test publication bias. Subgroup analyses were carried out for reintervention for Asian and Non-Asian populations, aged <18 group, and type I SVAS group. Sensitivity analysis was conducted to assess robustness of the results. Statistical significance was set at *p* < 0.05. All the analyses were performed with R (v 4.0.3).

## Results

### Eligible studies

**Figure 1:**
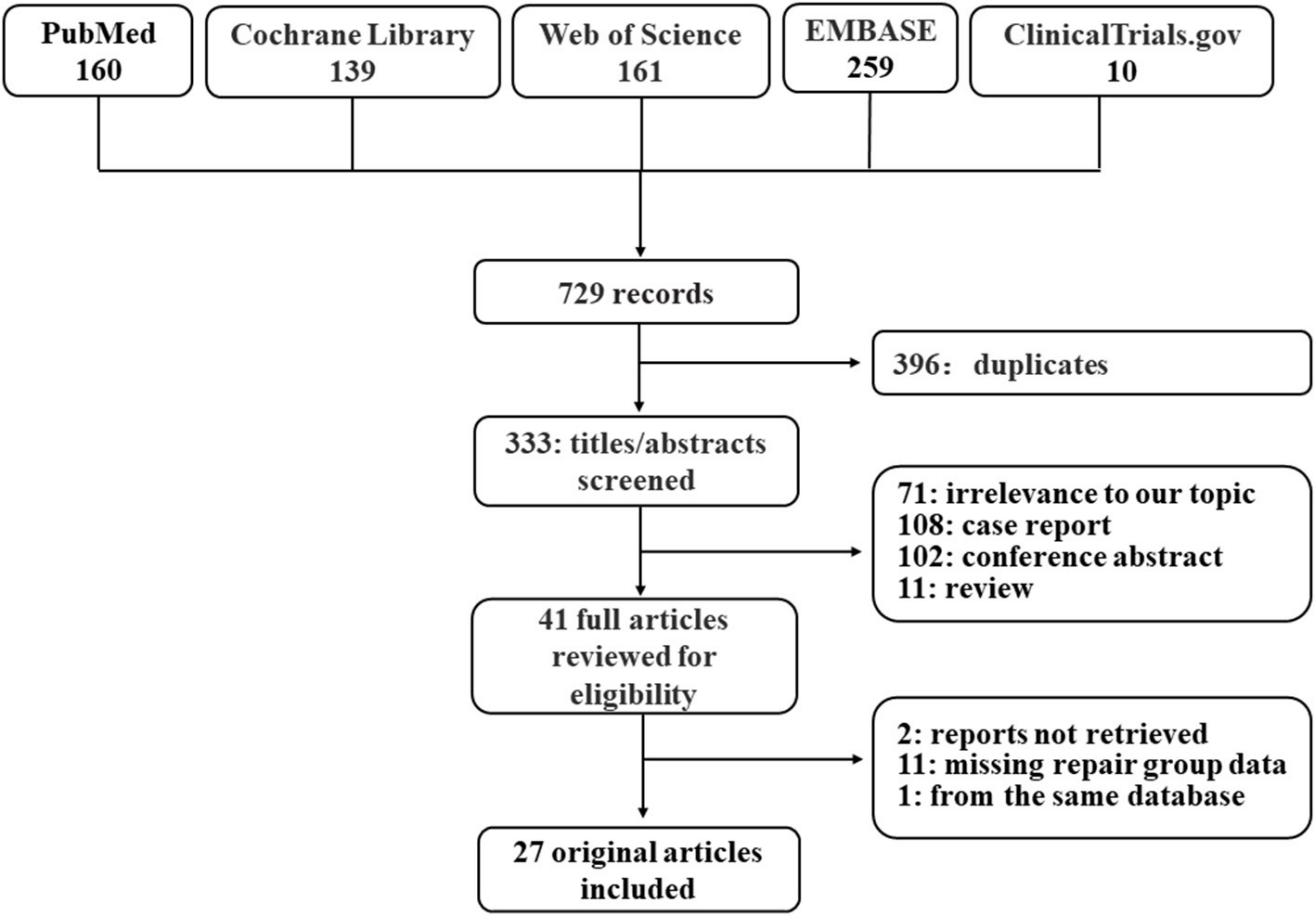
PRISMA flow chart for study selection.

The process of the literature retrieval was illustrated in Figure 1. A total of 729 citations were obtained by searching PubMed, Cochrane Library, Web of Science, EMBASE, and ClinicalTrials.gov, of which 333 records remained after 396 duplicates had been removed. After reviewing titles and abstracts, 41 citations remained for the full-text screening and 71 records were deleted due to irrelevance to our study. Finally, 27 studies (7, 8, 11, 14, 16–22, 30–45) were included.

### Study characteristics

Table 1 shows the characteristics of 27 eligible cohorts constituted of 1,162 patients, with 542 (46.6%) undergoing single-patch, 394 (33.9%) two-patch, and 226 (19.4%) three-patch. Among these cohorts, 17 were comparative studies reporting at least two repair methods, 2 studies (8, 40) compared three-patch method with another method without separated results of single- or two-patch, and 8 studies were single group. The mean ages of the participants ranged from 3.0- to 15.8-year-old. The mean follow-up time ranged from 1.2 to 8.0 years. The loss of follow-up ranged from 0 to 26.7%.

**Table 1:** Baseline information of the included studies.

**First Author (Year), Country**	**Group**	***N***	**Age (y)^a^**	**Women *N* (%)**	**Type II *N* (%)**	**Williams *N* (%)**	**PA stenosis *N* (%)**	**Patch material (Pericardium) *N* (%)**	**Pre-gradient (mmHg)**	**Follow-up time (y)**
**Comparison studies**										
Ibarra (2021), USA (30)	Total	89	2.5 (1.0, 6.8)	57 (64)	15 (20)	40 (45)	19 (21)			5.8 (1.8,10.7)
	Single	31	5.1 (0.9, 8.2)		11 (35)	6 (19)	2 (6)			5.1 (1.0, 9.9)
	Two	58	2.5 (1.0, 5.7)		4 (7)	34 (59)	17 (29)			5.9 (2.9, 11.5)
Hu (2021), China (19)	Total	225	2.2 (1.2, 4.4)	83 (36.9)	66 (29.3)				69.0 (51.0, 93.0)	3.7 (1.9, 5.7)
	Single	178	2.2 (1.3, 4.4)	69 (38.8)	52(29.2)				66.0 (49.3, 97.3)	3.4 (1.8, 5.3)
	Two	44	2.3 (1.2, 3.8)	12 (27.3)	13 (29.6)				81.0 (60.8, 97.3)	5.0 (2.8, 7.4)
	Three	3	4.8 (2.7, 5.3)	2 (66.7)	1 (33.3)				76.0 (56.0, 93.0)	8.8 (7.8, 8.8)
Biçer (2021), Turkey (31)	Total	29	4.5 (3.0, 9.9)	12 (41.4)	24 (82.8)	10 (34.5)		23 (79.3)	90 (75, 110)	2.5 (0.7, 7.3)
	Single	14	4.6 (2.2, 8.3)	7 (50.0)	9 (64.3)	6 (42.9)		12 (80)	90 (79, 110)	4.9 (1.6, 7.3)
	Two	15	6.5 (3.6, 13.3)	5 (33.3)	15 (100.0)	4 (26.7)		11 (78.6)	87 (75, 121)	1.9 (0.1, 7.6)
Wu (2019), USA (14)	Total	83	2.9 (0.8, 6.3)	36 (43.3)	39 (47.0)			69 (79.3)		3.7
	Single/two/three *N* = 9/24/50									
Peng (2019), China (20)	Total	91	10.0 ± 2.5	26 (28.6)				59 (64.8)	74.30 ± 35.67	1.25
	Single/two/three *N* = 29/47/15									
Roemers (2017), Netherlands (16)	Total	49	6 (2.0, 11)	27 (55.1)	7 (14.3)	24 (49.0)		35 (71.4)		Median 19
	Single	11			0 (0.0)					
	Two	12			0 (0.0)					
	Three	26	4 (2.0, 9.0)	13 (50.0)	7 (26.9)	19 (73.1)				
Liu (2017), China (32)	Total	90	3.0 ± 2.5	34 (37.8)	39 (43.3)		71 (78.9)	30 (33.3)	75.5 ± 35.8	3.2 ± 1.2
	Single	63			0 (0.0)					
	Two	24			0 (0.0)					
	Three	3			0 (0.0)					
Fricke (2014), Australia (33)	Total	28	5.2 (0.3, 13.1)	10 (36)	4 (14.3)	28 (100.0)	13 (46.4)	0 (0.0)	71 + 19	11.2 ± 4.5
	Single	1	0.6		0 (0.0)	1 (100.0)	1 (100.0)			
	Two	17	5.1 (0.3, 13.1)		3 (17.6)	17 (100.0)	10 (58.8)			14.7 ± 4.5
	Three	10	5.2 (3.4, 11.1)		1 (10.0)	10 (100.0)	2 (20.0)			5.0 ± 2.4
Kramer (2014), Germany (34)	Total	38	3.3 ± 8.6	13 (34.2)	5 (13.2)	20 (52.6)	17 (44.7)		86.1 ± 28.7	Median 7.5
	Single	3	6.8 ± 6.0						59.7 ± 25.9	
	Two	22	12.7 ± 16.0						87.9 ± 32.0	
	Three	13	3.4 ± 3.0						89.2 ± 21.3	
Kasnar-Samprec (2012), Germany (35)	Total	26	8.8 ± 4.3	8 (30.8)	6 (23)	17 (65)	10 (39)		73 ± 27	Median 17.7
	Single	16								
	Two	10								
Kaushal (2010), USA (36)	Total	20	Median 1.4	6 (30)	8 (40)	10 (50)			82 ± 17	
	Single	8	Median 1.1		4 (50.0)	2 (25.0)				10.0 ± 6.9
	Two	4	Median 1.0		2 (50.0)	2 (50.0)				4.0 ± 6.0
	Three	8	Median 1.9		6 (75.0)	6 (75.0)				3.8 ± 3.2
Scott (2009), USA (8)	Total	25	6.4 ± 5.7		7 (28.0)	21 (84.0)			81 ± 23	6.3 ± 4.5
	Single/two	15	5.9 ± 4.9		6 (40.0)	14 (93.3)			80 ± 26	7.0 ± 6.4
	Three	10	7.5 ± 6.8		1 (10.0)	7 (70.0)			81 ± 20	5.1 ± 3.5
Metton (2009), france (18)	Total	34	5.5 ± 6	12(35)	10(29)	14(41)	15(44)		104 ± 21	5.8 ± 1.9
	Single	8/3/23								
	Two	3								
	Three	23								
Koçyildirim (2009), turkey (37)	Total	25	4.4 ± 2.5	11 (44.0)	0 (0.0)	12 (48.0)	9 (36.0)		65.1 ± 12.9	6.8 ± 2.7
	Single	14	3.9 ± 1.4	9 (64.3)	0 (0.0)	7 (50.0)	5 (35.7)		72.61 ± 16.20	7.07 ± 3.61
	Three	11	5.2 ± 3.5	5 (45.5)	0 (0.0)	5 (45.5)	4 (36.4)		57.58 ± 9.54	6.55 ± 3.61
Brown (2002), India (21)	Total	101	6.1 ± 2.8	40 (39.6)	28 (27.7)	14 (13.9)			90 ± 33	Medium 9.4
	Single	85			14 (16.5)	0 (0.0)				
	Two	12			12 (100.0)	9 (75.0)				
Hazekamp, (1999), Netherlands (39)	Total	29	15.8 ± 12.5	15 (51.7)	4 (13.8)	11 (37.9)	3 (10.3)	29 (100.0)	84 ± 37	10.9 ± 8.1
	Single	14						14 (100.0)		
	Two	2						2 (100.0)		
	Three	13						13 (100.0)		
Stamm (1999), USA (38)	Total	75	7.4 ± 4.4	39 (52.0)	19 (25.3)	46 (61.3)	31 (41.3)		86 ± 29	12.8 ± 6.2
	Single	34			9 (26.5)					
	Two	35			10 (28.6)					
	Three	6								
Minakata (1997), Japan (22)	Total	8	7.3 ± 2.0	3 (37.5)	1 (12.5)	6 (75.0)		0 (0.0)	68.0 ± 14.2	8.0 ± 2.6
	Single	2	6 ± 2.8	0 (0.0)	0 (0.0)	2 (100.0)		0 (0.0)	43 ± 32.5	15.6 ± 2.4
	Two	6	7.2 ± 4.2	3 (50.0)	1 (16.7)	4 (66.7)		0 (0.0)	76.5 ± 19.6	5.5 ± 4.4
Myers (1993), USA (40)	Total	13	7.6 ± 3.8	7 (53.8)		4 (30.8)		4 (30.8)	64.5 ± 23.6	3.8 ± 4.0
	Single/two	7				3 (75.0)	2 (50.0)	0 (0.0)	73 ± 24.0	6.0 ± 4.5
	Three	6				1 (16.7)	4 (66.7)	4 (66.7)	54.5 ± 20.6	1.2 ± 0.6
**Single group studies**										
Monge (2017), USA (17)	Three	20	3.7 ± 5.9	7 (35.0)	6 (30.0)	12 (60.0)	10 (50.0)	1 (5.0)	78.4 ± 29.1	Median 4.0
Işik (2017), Turkey (41)	Two	10	4.8 ± 3.9	4 (40.0)	1 (10.0)	4 (40.0)	2 (20.0)		90 ± 25.5	3.7 ± 1.2
Bakhtiary (2013), Germany (42)	Two	21	3.1 ± 4.2	6 (29)		14 (66.7)		21 (100)	77 ± 34	4.3 ± 2.9
Kavarana (2012), USA (43)	Single	22	2.4 ± 2.4	8 (36.4)	5 (22.73)	10 (45.5)	11 (50)		77.23 ± 26.87	Median 2.7
Cruz-Castañeda (2009), Mexico (44)	Three	9	9.4 ± 3.4	7 (77.8)	0 (0.0)	8 (88.9)		6 (66.7)	51.3 ± 17.3	1.5 ± 0.7
Delius (1995), USA (7)	Two	15	6.5 ± 1.9	3 (20)	1 (6.7)			0 (0.0)	91.0 ± 15.8	Median 11.75
Stewart (1988), USA (45)	Two	5	24.4 ± 26.1	2 (40.0)	1 (20.0)	4 (80.0)		0 (0.0)	104.6 ± 18.6	0.5–10
Doty (1977), USA (11)	Two	8	8.5 ± 3.5	3 (37.5)	2 (25.0)	1 (12.5)	1 (12.5)	0 (0.0)	91.6 ± 25.7	11.8 ± 7.6

a mean ± SD/median (IQR).

PA, pulmonary artery; USA, the United States of America.

For the total populations, 472 (41.0%) were women, 291 (28%) were Type I SVAS, 305 (47.2%) were WBS, 212 (42.9%) had PAS, and the mean of pre-operation gradient was 80.9 mmHg. For 18 studies reported group baseline information, patient underwent two-patch and three-patch repair were more likely to be WBS [(34 (19.2%) for one-patch, 93 (59.6%) for two-patch, and 68 (68.0%) for three-patch)], had PAS [(19 (27.9%) for one-patch, 30 (32.3%) for two-patch and 20 (42.6%) for three-patch)] and had a higher pre-operation gradient (mean: 68.1 mmHg for one-patch, 87.4 mmHg for two-patch and 69.7 mmHg for three-patch).

### Risk of bias assessment

The mean MINORS score of 19 comparative studies was 15.8, ranging from 13 to 20 and the mean MINORS score of 8 non-comparative studies was 10.9, ranging from 9 to 12. All studies reported clearly stated aims, appropriate endpoints, follow-up periods, adequate control group, and contemporary groups. However, none of the studies had prospective data collection and calculation of study size (Supplementary Table S2).

### Main network meta-analyses

**Figure 2:**
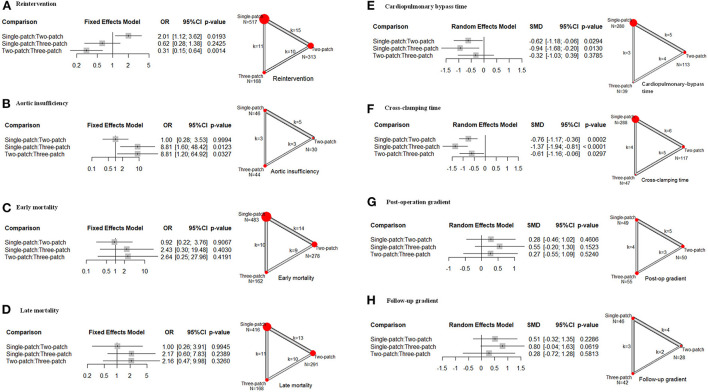
Effect size of the following outcomes for supravalvar aortic stenosis *via* the three corrective surgeries derived from the network meta-analysis. **(A)** Reintervention; **(B)** aortic insufficiency; **(C)** early mortality; **(D)** late mortality; **(E)** cardiopulmonary bypass time; **(F)** cross-clamping time; **(G)** post-operation transvalvular pressure gradient; and **(H)** transvalvular pressure gradient at follow-up. The width of the lines represents the number of studies comparing each pair of treatments. The size of the red point represents the sample size in each arm. OR, odds ratio; SMD, standardized mean difference; 95% CI, 95% confidence interval.

Two-patch repair had a lower rate of reintervention compared with single-patch (15 studies, OR = 0.47, 95 % CI 0.28–0.89), or three-patch (10 studies, OR = 0.31, 95 % CI 0.15–0.64) (Figure 2A). And single-patch had no statistical difference in reintervention rate compared with three-patch (11 studies, OR = 0.62, 95 % CI 0.28–1.38). No heterogeneity was found for the reintervention rate among the studies (τ^2^ = 0.288, *I*^2^ = 14.3%, Q statistic =28.00, *p* = 0.260). Multiple pairwise meta-analyses gave the similar results (Supplementary Table S3; Supplementary Figure S1A). Three-patch repair had a lower rate of aortic insufficiency compared with single-patch (3 studies, OR = 0.11, 95 % CI 0.01–0.63), and two-patch (3 studies, OR = 0.11, 95 % CI 0.02–0.83) (Figure 2B). Single-patch had no statistical difference with two-patch on aortic insufficiency rate (5 studies, OR = 1.00, 95 % CI 0.28–3.53). No heterogeneity was found for the reintervention rate among the studies (τ^2^ = 0, *I*^2^ = 0%, Q statistic = 1.31, *p* = 0.971). Multiple pairwise meta-analyses only pointed out that three-patch had a lower rate compared with single-patch (Supplementary Table S3; Supplementary Figure S1B). There was no statistically significant difference in early mortality, late mortality, post-operation gradient, and follow-up gradient (Figure 2).

As for the operation time, owing to the heterogeneity (CPB: τ^2^ = 0.285, *I*^2^ = 68.3%, Q statistic =22.10, *p* < 0.05; ACC: τ^2^ = 0.126, *I*^2^ = 45.2%, Q statistic =16.43, *p* = 0.058), random effects models were conducted. Single-patch method had a shorter CPB time and CCP time than two-patch (CPB: 5 studies, SMD = −0.62, 95 % CI −1.18 to −0.06; CCP: 6 studies, SMD = −0.76, 95 % CI −1.17 to −0.36), and three-patch (CPB: 3 studies, SMD = −0.94, 95 % CI −1.68 to −0.20; CCP: 5 studies, SMD = −0.61, 95 % CI −1.16 to −0.06) (Figures 2E,F). Two-patch had no statistical difference compared with three-patch on CPB time (4 studies, SMD = −0.32, 95 % CI −1.03 to 0.39), but two-patch had shorter CCP time than three-patch (5 studies, SMD = −0.61, 95 % CI −1.16 to −0.06) (Figure 2F). The multiple pairwise meta-analyses gave the similar results (Supplementary Table S3; Supplementary Figures S1E,F). No publication bias was found among all these outcomes.

### Subgroup analysis

**Figure 3:**
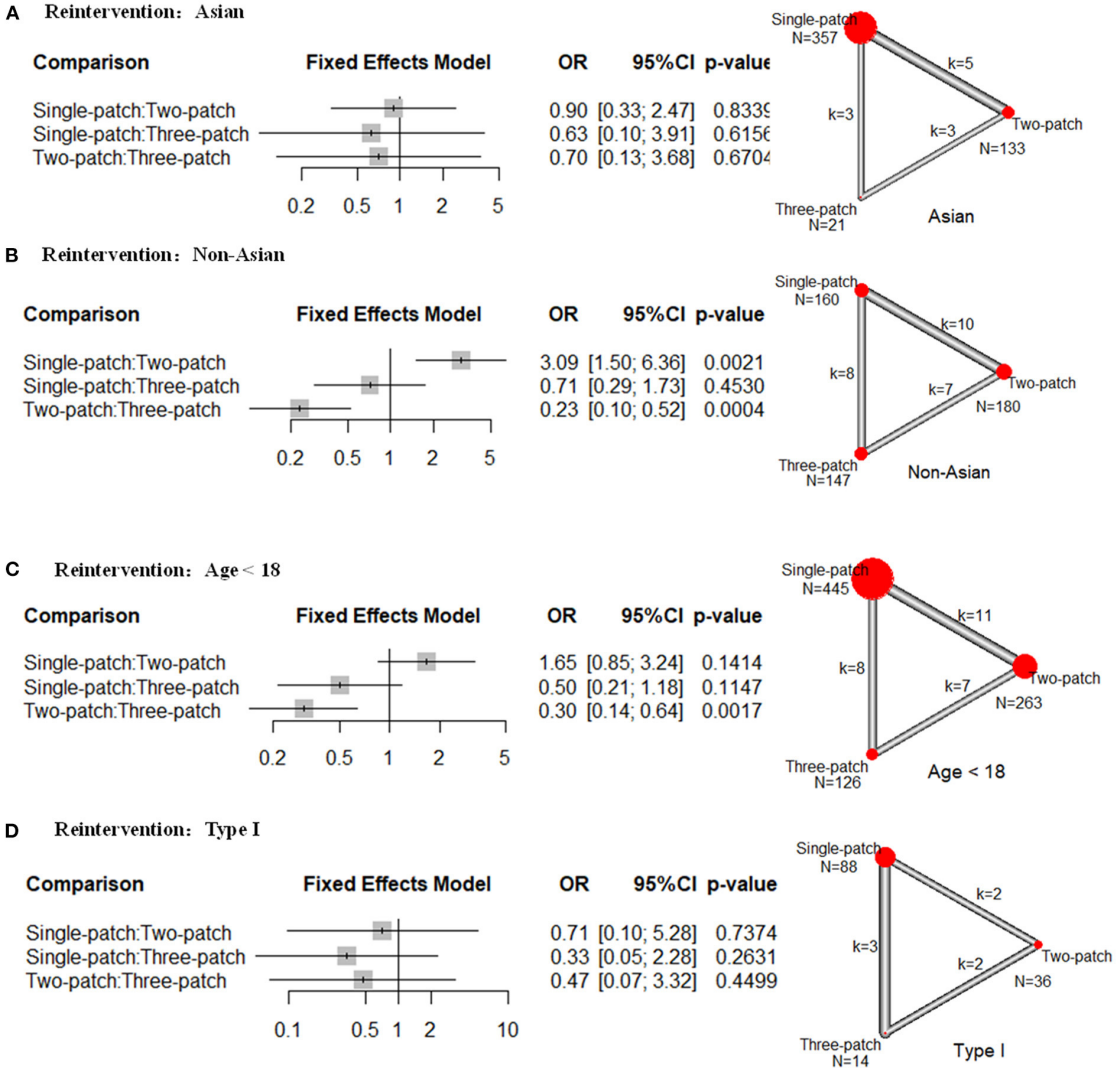
Subgroup analysis of odds ratios (OR) of the following outcomes for supravalvar aortic stenosis *via* the three corrective surgeries derived from the network meta-analysis. **(A)** reintervention rates in Asian regions; **(B)** reintervention rates in non-Asian regions; **(C)** reintervention rates for patients younger than 18 years; and **(D)** reintervention rates for patients with type I supravalvar aortic stenosis. The width of the lines represents the number of studies comparing each pair of treatments. The size of the red point represents the sample size in each arm.

There was no statistical difference in the reintervention rate for the Asian population among single-, two-, and three-patch (Figure 3A). For Non-Asian populations, two-patch repair had a lower rate compared with single-patch (10 studies, OR = 0.32, 95 % CI 0.16–0.67), or three-patch (7 studies, OR = 0.23, 95 % CI 0.10–0.52) (Figure 3B). And single-patch had no statistical difference in reintervention rate compared with three-patch (8 studies, OR = 0.71, 95 % CI 0.29–1.73). For age <18 group, two-patch repair had a lower rate of reintervention than three-patch (7 studies, OR = 0.30, 95 % CI 0.14–0.64) (Figure 3C). And single-patch had no statistical difference compared with two-patch (11 studies, OR = 1.65, 95 % CI 0.85–3.24) or three-patch (8 studies, OR = 0.50, 95 % CI 0.21–1.18). There was no statistical difference in reintervention rate for Type I SVAS among single-, two-, and three-patch (Figure 3D). No heterogeneity was found in the subgroup analysis.

## Discussion

Our review aimed to answer two patient-oriented questions that are closely related to clinical practice: (1) which surgical procedure (single-, two- or three-patch) had a better prognosis for patients with SVAS? And (2) how could clinicians best treat juvenile and adult patients with different types of SVAS? The results of the network meta-analysis indicated that two-patch repair had the lowest reintervention rate compared with the single- or three-patch repair. This finding also applied to juvenile and non-Asian patients. Although three-patch repair had a lower risk of aortic insufficiency, this repair had the longest CCP time, which was significantly longer than that of single- or two-patch repair.

Our meta-analysis also found that the surgical procedures chosen for SAVS were most frequent in single-patch (46.6%), then two-patch (33.9%), and least frequent in three-patch (19.4%). Two- and three-patch repair had a higher proportion of patients who were WBS, had PAS or higher preoperative pressure gradient compared with single-patch. This indicated that patients with more sites of preoperative stenosis accumulation or more severe stenosis were more inclined to apply two-patch or three-patch for correction, similar to the conclusions reached by Kramer et al. (34), patients treated with the two- and three-patch repair had a combination of more cardiovascular anomalies and higher preoperative pressure gradients.

For patients with SVAS, reinterventions were mainly caused by recurrent supravalvular stenosis, distal residual stenosis, aortic insufficiency, and PAS (14–16, 19, 20, 30, 31, 33, 35, 36, 43). The common shape in two-patch repair is an inverted pantaloon-shaped, which widens the aortic root in the non-coronary sinus and the right sinus. Two-patch repair was first proposed by Doty (11) in 1977. This method is more suitable for severe SVAS than single-patch repair because the latter failed to allow the supravalvular fibrous ring to be very thick and very rigid aortic opening wide. In addition, the aortic valve cusps may obstruct the coronary artery orifice after asymmetric reconstruction. Also, two-patch repair is easier to perform and less time-consuming (CPB and CCP) compared with three-patch repair. Although the three-patch repairs do restore aortic root symmetry, they also increase the time of surgery in patients with severe left ventricular hypertrophy, placing them at an increased propensity for subendocardial ischemia and tenuous myocardial protection (36). Hence, two-patch repair had some advantages over other repairs, such as adequately widening the STJ of severe obstruction, relatively symmetrical reconstruction of the aortic root preserving the function of the aortic valve, and a simple operation (less time-consuming), making the application of the procedure more reproducible and overall reintervention rate much lower.

For the Asia group, no significant difference was observed for the reason that only 21 patients were treated with three-patch repair, which caused a wide 95% confidence interval. For type I SVAS, no significant difference was found among these surgical approaches because type I SVAS tends to be a milder condition that is easier to treat. As suggested by Koçyildirim et al. (37), the application of a single-patch for type I (discrete) SVAS was easy, safe, and apparently durable. We did not perform analyses in adults and type II subgroups due to a lack of adequate studies. Considering that the main populations of congenital heart disease remain pediatric patients, the advantage of two-patch repair in terms of reintervention rates in patients younger than 18 years is worthy of our attention.

However, three-patch exhibited a lower incidence of postoperative aortic insufficiency compared with two-patch, inconsistent with the results of reintervention. The possible reason was the heterogeneity from the study of Metton et al. (18). After removing this study, the results of the network meta-analysis found no significant difference between three-patch and two-patch repair, and were same with the pairwise meta result. Although no significant differences were found for the postoperative and follow-up pressure gradients, the point estimation values of the postoperative and follow-up pressure gradients were lower in two-patch and three-patch groups than the single-patch group, and the wide confidence intervals were caused by the insufficient number of studies. Stamm et al. (38) had also shown that the application of multi-sinus correction of SVAS was more effective than single-patch correction in reducing pressure gradients, for the reason that multi-sinus correction was more effective in relieving obstruction and widening the outflow tract to restore the aortic anatomy.

## Study bias

The eligible studies were not prospective cohort or randomized controlled trials. The baseline information of most studies was not fully balanced, and all studies did not use multivariate analysis, which could cause the bias. Because patients with two- or three-patch repair had more severe symptoms, we might underestimate the effect of two- or three-patch repair. Since no eligible study calculated the sample size, the study power could not be reached, and the insufficient events might cause a wide confidence interval (46).

## Strengths and limitations

This study was the first network meta-analysis that systematically compared three surgical procedures for the correction of SVAS and comprehensively included relevant studies based on 5 databases. However, some limitations could not be ignored. First, some studies did not report the required baseline and outcome information. Although we had sent emails to the authors, only a few replied. Thus, subgroup analysis was limited and meta-regression analysis could not be performed. But given the relatively low heterogeneity of outcomes in our study, it is not necessary to conduct the meta-regression analysis. Second, the lack of operator information and the differences in treatment levels among operators and cardiac centers made the results of some studies biased. Third, the included studies were all traditional three procedures and did not include the results of new and improved procedures (47, 48), which may have biased the results to some extent. Finally, the range of the publication year of the included studies was very wide, but the analysis didn't observe heterogeneity of publication years. SVAS is a rare disease, and it needs all the relevant studies to make sure the sufficient cases.

## Future prospective

Patch materials influence the occurrence of restenosis and aortic insufficiency, but the selection of patch materials varied in operators and hospitals (16, 49–52). Future studies should be adequately designed to apply several clinically validated patch materials for postoperative efficacy analysis. And studies should also balance the operator's surgical experience.

## Conclusion

Two-patch repair had the lowest reintervention rate and relatively reasonable operation time. Complex and severe SVAS is recommended to be treated with two-patch repair. Prospective studies of reasonable sample size will be required with a special focus on the use of different patch materials and surgeons' diverse working experience.

## Data availability statement

The original contributions presented in the study are included in the article/Supplementary material, further inquiries can be directed to the corresponding author.

## Author contributions

LL and XL designed the study, collected and analyzed the data, and wrote the manuscript. SZ, CW, and QW revised the manuscript. All authors contributed to the article and approved the submitted version.

## Funding

The study was supported by the Central Public-interest Scientific Institution Basal Research Fund (2019XK320050), Central University Basic Research Fund (APL20100410010302004), and Yunnan Provincial Cardiovascular Disease Clinical Medical Center Project (FZX2019-06-01).

## Conflict of interest

The authors declare that the research was conducted in the absence of any commercial or financial relationships that could be construed as a potential conflict of interest.

## Publisher's note

All claims expressed in this article are solely those of the authors and do not necessarily represent those of their affiliated organizations, or those of the publisher, the editors and the reviewers. Any product that may be evaluated in this article, or claim that may be made by its manufacturer, is not guaranteed or endorsed by the publisher.
