# Supplementary material for: Assessment of three types of surgical procedures for supravalvar aortic stenosis: A systematic review and meta-analysis
Source: Front Cardiovasc Med. 2022 Sep 6;9:987522. doi: 10.3389/fcvm.2022.987522 (PMC9485597; doi:10.3389/fcvm.2022.987522)
Supplement: Supplementary file 1 [file Data_Sheet_1.DOCX]

**Assessment of three types of surgical procedures for supravalvar aortic stenosis: A** **Systematic Review and Meta-Analysis**

**(Supplementary appendix)**

**Supplementary Methods.** Search strategy
**Table S1.** Outcomes of the included studies

**Table S2.** Quality assessment of the included studies

**Table S3.** Results from multiple pairwise meta-analyses (Direct comparison)

**Figure S1.** Forest plots and funnel plots for the multiple pairwise meta-analyses

**Figure S2.** Sensitive analysis

**Supplementary Methods.** Search strategy

**PUBMED**

((McGoon) OR (Doty) OR (Brom) OR (sliding) OR (sinus) OR (repair) OR (patch)) AND (supravalvular aortic stenosis)

((McGoon[Title/Abstract]) OR (Doty[Title/Abstract]) OR (Brom[Title/Abstract]) OR (sliding[Title/Abstract]) OR (sinus[Title/Abstract]) OR (repair[Title/Abstract]) OR (patch[Title/Abstract])) AND (supravalvular aortic stenosis[Title/Abstract])

**Cochrane Library Trials**

((McGoon) OR (Doty) OR (Brom) OR (sliding) OR (sinus) OR (repair) OR (patch)) AND (aortic stenosis) in Title Abstract Keyword - (Word variations have been searched)

**Web of science**

((McGoon) OR (Doty) OR (Brom) OR (sliding) OR (sinus) OR (repair) OR (patch)) AND (supravalvular aortic stenosis)

**EMBASE**

((McGoon) OR (Doty) OR (Brom) OR (sliding) OR (sinus) OR (repair) OR (patch)) AND (supravalvular aortic stenosis):ab,ti,kw

**ClinicalTrials.gov**

supravalvular aortic stenosis

**Table S1. Outcomes of the included studies**

| Author, Year | group | N | Aortic insufficiency N (%) | Reintervention N (%) | Early mortality  N (%) | Late mortality  N (%) | CBP, min | ACC, min | Post-op gradient, mmHg | Follow-up gradient, mmHg |
| --- | --- | --- | --- | --- | --- | --- | --- | --- | --- | --- |
| Comparison studies | | | | | | | | | | |
| Ibarra, 2021 | single | 31 |  | 8 (25.8) | 0 (0.0) | 1 (3.2) |  |  |  |  |
|  | two | 58 |  | 4 (6.9) | 0 (0.0) | 0 (0.0) |  |  |  |  |
| Hu, 2021 | single | 178 |  | 9 (5.1) | 5 (2.8) | 2 (1.1) | 75.5 ± 38.7 | 45.0 ± 23.4 |  |  |
|  | two | 44 |  | 2 (4.5) | 2 (4.5) | 1 (2.3) | 111.0 ± 30.2 | 70.5 ± 28.5 |  |  |
|  | three | 3 |  | 0 (0.0) | 0 (0.0) | 0 (0.0) | 140.0 ± 50.8 | 75.0 ± 14.1 |  |  |
| Biçer, 2021 | single | 14 | 7 (50.0) | 3 (21.4) | 0 (0.0) | 0 (0.0) | 123.0 ± 79.3 | 56.0 ± 35.6 | 17 ± 21.6 | 7 ± 5.9 |
|  | two | 15 | 8 (53.3) | 0 (0.0) | 1 (6.7) | 0 (0.0) | 77.0 ± 90.4 | 51.0 ± 55.6 | 11 ± 8.2 | 5 ± 3.7 |
| Wu, 2019 | single | 9 |  | 3 (33.3) | 0 (0.0) | 1 (11.1) |  |  |  |  |
|  | two | 24 |  | 8 (33.3) | 0 (0.0) | 0 (0.0) |  |  |  |  |
|  | three | 50 |  | 37 (74.0) | 0 (0.0) | 2 (4.0) |  |  |  |  |
| Peng, 2019 | single | 29 |  | 1 (3.4) | 0 (0.0) | 0 (0.0) |  |  |  |  |
|  | two | 47 |  | 5 (10.6) | 4 (8.5) | 0 (0.0) |  |  |  |  |
|  | three | 15 |  | 2 (13.3) | 0 (0.0) | 0 (0.0) |  |  |  |  |
| Roemers, 2017 | single | 11 |  | 1 (9.1) | 1 (9.1) | 2 (18.2) |  |  |  |  |
|  | two | 12 |  | 1 (8.3) | 0 (0.0) | 2 (16.7) |  |  |  |  |
|  | three | 26 |  | 6 (23.1) | 1 (3.8) | 2 (7.7) |  |  |  |  |
| Liu, 2017 | single | 63 |  | 1 (1.6) | 3 (4.8) | 0 (0.0) | 85.4 ± 49.8 | 45.1 ± 23.3 |  |  |
|  | two | 24 |  | 1 (4.2) | 0 (0.0) | 1 (4.2) | 107.0 ± 43.7 | 60.0 ± 19.9 |  |  |
|  | three | 3 |  | 0 (0.0) | 0 (0.0) | 0 (0.0) | 138.7 ± 69.5 | 74.3 ± 19.0 |  |  |
| Fricke, 2014 | single | 1 |  | 1 (100.0) | 0 (0.0) | 0 (0.0) | 347 | 180 |  |  |
|  | two | 17 |  | 5 (29.4) | 0 (0.0) | 4 (23.5) | 114.0 ± 126.0 | 65.0 ± 90.4 |  |  |
|  | three | 10 |  | 0 (0.0) | 0 (0.0) | 0 (0.0) | 116.0 ± 83.0 | 92.0 ± 43.7 |  |  |
| Kramer, 2014 | single | 3 |  |  |  |  |  |  | 19.7 ± 9.0 |  |
|  | two | 22 |  |  |  |  |  |  | 21.4 ± 11.1 |  |
|  | three | 13 |  |  |  |  |  |  | 24.5 ± 12.0 |  |
| Kasnar-Samprec, 2012 | single | 16 |  | 2 (12.5) | 3 (18.8) |  | 50 ± 24 | 31 ± 15 |  |  |
|  | two | 10 |  | 2 (20.0) | 0 (0.0) |  | 95 ± 27 | 52 ± 16 |  |  |
| Kaushal, 2010 | single | 8 | 4 (50.0) | 5 (62.5) | 1 (12.5) | 0 (0.0) |  | 40 ± 14 | 31 ± 12 | 33 ± 18 |
|  | two | 4 | 0 (0.0) | 0 (0.0) | 0 (0.0) | 0 (0.0) |  | 60 ± 39 | 17 ± 9.7 | 10 ± 1 |
|  | three | 8 | 1 (12.5) | 0 (0.0) | 0 (0.0) | 0 (0.0) |  | 104 ± 21 | 22 ± 15 | 18 ± 12 |
| Metton, 2009 | single | 8 | 3 (37.5) | 1 (12.5) | 0 (0.0) | 0 (0.0) | 90 ± 43 | 47 ± 25 | 37.5 ± 17 | 45 ± 28 |
|  | two | 3 | 1 (33.3) | 0 (0.0) | 0 (0.0) | 0 (0.0) | 106 ± 29 | 58 ± 9 | 30 ± 15 | 30 ± 9 |
|  | three | 23 | 1 (4.3) | 0 (0.0) | 1 (4.3) | 0 (0.0) | 114 ± 47 | 65 ± 17 | 14 ± 10 | 11 ± 18 |
| Koçyıldırım, 2009 | single | 14 |  | 2 (14.3) | 0 (0.0) | 0 (0.0) |  |  | 14.76 ± 7.22 | 15.39 ± 4.54 |
|  | three | 11 |  | 0 (0.0) | 0 (0.0) | 0 (0.0) |  |  | 13.48 ± 6.77 | 14.96 ± 5.03 |
| Brown, 2002 | single | 85 |  | 13 (15.3) | 3 (3.5) |  |  |  |  |  |
|  | two | 12 |  | 1 (8.3) | 0 (0.0) |  |  |  |  |  |
| Hazekamp, 1999 | single | 14 | 0 (0.0) | 1 (7.1) | 1 (7.1) | 1 (7.1) |  |  |  |  |
|  | two | 2 | 0 (0.0) | 0 (0.0) | 0 (0.0) | 0 (0.0) |  |  |  |  |
|  | three | 13 | 0 (0.0) | 1 (7.7) | 0 (0.0) | 1 (7.7) |  |  |  |  |
| Stamm, 1999 | single | 34 |  | 12 (35.3) |  | 7 (20.6) |  |  |  |  |
|  | two | 35 |  | 2 (5.7) |  | 0 (0.0) |  |  |  |  |
|  | three | 6 |  | 0 (0.0) |  | 0 (0.0) |  |  |  |  |
| Minakata, 1997 | single | 2 | 0 (0.0) | 1 (50.0) | 0 (0.0) | 1 (50.0) |  |  | 12.5 ± 10.6 | 11 ± 7.1 |
|  | two | 6 | 0 (0.0) | 0 (0.0) | 0 (0.0) | 0 (0.0) |  |  | 27 ± 13.8 | 25.3 ± 31.1 |
| Single group studies | | | | | | | | | | |
| Monge, 2017 | three | 20 | 2 (10.0) | 0 (0.0) | 0 (0.0) | 0 (0.0) | 172 ± 29 | 110 ± 21 | 17.4 ± 21.0 | 13.9 ± 20.4 |
| Işık, 2017 | two | 10 | 0 (0.0) | 0 (0.0) | 1 (10.0) | 1 (10.0) | 79.7 ± 20.5 | 57.6 ± 18.2 | 20 ± 8.4 | 14 ± 4.2 |
| Bakhtiary, 2013 | two | 21 |  | 0 (0.0) | 0 (0.0) | 1 (4.8) | 112 ± 76 | 44 ± 24 | 20 ± 10 | 15 ± 8 |
| Kavarana, 2012 | single | 22 | 0 (0.0) | 2 (9.1) | 0 (0.0) |  | 79.55 ±47.47 | 40 ± 28.03 | 18.19 ± 18.17 | 10 ± 12 |
| Scott, 2009 | three | 10 | 0 (0.0) | 0 (0.0) | 1 (10.0) | 0 (0.0) | 121 ± 93 | 54 ± 15 | 15 ± 7 | 15 ± 13 |
| Cruz-Castañeda, 2009 | three | 9 |  | 0 (0.0) | 1 (11.1) |  |  |  | 7.8 ± 7.5 |  |
| Delius, 1995 | two | 15 | 9 (60.0) | 6 (40.0) | 0 (0.0) | 2 (13.3) |  |  | 23 ± 8.3 | 36 ± 15 |
| Myers, 1993 | three | 6 | 0 (0.0) | 0 (0.0) | 1 (16.7) |  |  |  | 12.2 ± 10.4 |  |
| Stewart, 1988 | two | 5 |  | 0 (0.0) | 1 (20.0) | 0 (0.0) |  |  | 15 ± 15 |  |
| Doty, 1977 | two | 8 | 1 (12.5) | 2 (25.0) | 0 (0.0) | 0 (0.0) |  |  | 28.1 ± 24.6 |  |

Abbreviations: ACC, aortic cross-clamp; CBP, cardiopulmonary bypass.

**Table S2.** Quality assessment of the included studies according to MINORS score

| ID | First Author | Year | Clearly stated aim | Inclusion of consecutive patients | Prospective data collection | Endpoints appropriate to study aim | Unbiased assessment of study endpoint | Follow-up period appropriate to study aim | <5% lost to follow-up | Prospective calculation of study size | Adequate control group | Contemporary groups | Baseline equivalence of groups | Adequate statistical analyses | Total |
| --- | --- | --- | --- | --- | --- | --- | --- | --- | --- | --- | --- | --- | --- | --- | --- |
| Comparative studies | | | | | | | | | | | | | | | |
| 1 | Ibarra | 2021 | 2 | 1 | 0 | 2 | 2 | 2 | 2 | 0 | 2 | 2 | 1 | 1 | 17/24 |
| 2 | Hu | 2021 | 2 | 2 | 0 | 2 | 2 | 2 | 2 | 0 | 2 | 2 | 2 | 2 | 20/24 |
| 3 | Biçer | 2021 | 2 | 2 | 0 | 2 | 2 | 2 | 1 | 0 | 2 | 2 | 2 | 0 | 17/24 |
| 4 | Wu | 2019 | 2 | 2 | 0 | 2 | 2 | 2 | 2 | 0 | 2 | 2 | 1 | 2 | 19/24 |
| 5 | Peng | 2019 | 1 | 2 | 0 | 2 | 2 | 2 | 2 | 0 | 2 | 2 | 0 | 0 | 15/24 |
| 6 | Roemers | 2017 | 2 | 1 | 0 | 2 | 2 | 2 | 2 | 0 | 2 | 2 | 1 | 1 | 17/24 |
| 7 | Liu | 2017 | 2 | 1 | 0 | 2 | 2 | 2 | 0 | 0 | 2 | 2 | 0 | 0 | 13/24 |
| 8 | Fricke | 2014 | 2 | 1 | 0 | 2 | 2 | 2 | 2 | 0 | 2 | 2 | 0 | 0 | 15/24 |
| 9 | Kramer | 2014 | 2 | 1 | 0 | 1 | 2 | 2 | 0 | 0 | 2 | 2 | 0 | 0 | 12/24 |
| 10 | Kasnar-Samprec | 2012 | 2 | 1 | 0 | 2 | 2 | 2 | 2 | 0 | 2 | 2 | 2 | 2 | 19/24 |
| 11 | Kaushal | 2010 | 2 | 1 | 0 | 2 | 2 | 2 | 2 | 0 | 2 | 2 | 0 | 2 | 17/24 |
| 12 | Scott | 2009 | 2 | 1 | 0 | 2 | 2 | 2 | 0 | 0 | 2 | 2 | 0 | 0 | 13/24 |
| 13 | Metton | 2009 | 2 | 1 | 0 | 2 | 2 | 2 | 2 | 0 | 2 | 2 | 0 | 0 | 15/24 |
| 14 | Koçyıldırım | 2009 | 2 | 1 | 0 | 2 | 2 | 2 | 2 | 0 | 2 | 2 | 2 | 1 | 18/24 |
| 15 | Brown | 2002 | 2 | 2 | 0 | 2 | 2 | 2 | 2 | 0 | 2 | 2 | 0 | 0 | 16/24 |
| 16 | Hazekamp | 1999 | 2 | 1 | 0 | 2 | 2 | 2 | 2 | 0 | 2 | 2 | 0 | 0 | 15/24 |
| 17 | Stamm | 1999 | 2 | 1 | 0 | 2 | 2 | 2 | 2 | 0 | 2 | 2 | 0 | 0 | 15/24 |
| 18 | Minakata | 1997 | 2 | 1 | 0 | 2 | 2 | 2 | 0 | 0 | 2 | 2 | 0 | 0 | 13/24 |
| 19 | Myers | 1993 | 2 | 1 | 0 | 2 | 2 | 2 | 0 | 0 | 2 | 2 | 0 | 0 | 13/24 |
| Non-comparative studies | | | | | | | | | | | | | | | |
| 1 | Monge | 2017 | 2 | 2 | 0 | 2 | 2 | 2 | 2 | 0 | NA | NA | NA | NA | 12/16 |
| 2 | Işık | 2017 | 2 | 1 | 0 | 2 | 2 | 2 | 2 | 0 | NA | NA | NA | NA | 11/16 |
| 3 | Bakhtiary | 2013 | 2 | 1 | 0 | 2 | 2 | 2 | 2 | 0 | NA | NA | NA | NA | 11/16 |
| 4 | Kavarana | 2012 | 2 | 1 | 0 | 2 | 2 | 2 | 2 | 0 | NA | NA | NA | NA | 11/16 |
| 5 | Cruz-Castañeda | 2009 | 2 | 1 | 0 | 2 | 2 | 2 | 2 | 0 | NA | NA | NA | NA | 11/16 |
| 6 | Delius | 1995 | 2 | 1 | 0 | 2 | 2 | 2 | 0 | 0 | NA | NA | NA | NA | 9/16 |
| 7 | Stewart | 1988 | 2 | 1 | 0 | 2 | 2 | 2 | 2 | 0 | NA | NA | NA | NA | 11/16 |
| 8 | Doty | 1977 | 2 | 1 | 0 | 2 | 2 | 2 | 2 | 0 | NA | NA | NA | NA | 11/16 |

Abbreviations: Methodological index for non-randomized studies (minors)

**Table S3. Results from multiple pairwise meta-analyses (Direct comparison)**

| Outcomes | No. of studies | OR/SMD (95%CI) | | Heterogeneity | | Egger test |
| --- | --- | --- | --- | --- | --- | --- |
|  |  | Fixed-effects models | Random-effects models | *I^2^* (%) | *p* value | *p* value |
| **Reintervention** | | | | | | |
| Single vs Two | 15 | 2.18 (1.31, 3.62) | 2.00 (1.03, 3.86) | 15.2 | 0.283 | 0.882 |
| Single vs Three | 11 | 1.15 (0.60, 2.17) | 1.25 (0.38, 4.14) | 51.4 | 0.024 | 0.036 |
| Two vs Three | 10 | 0.48 (0.25, 0.91) | 0.52 (0.20, 1.34) | 13.2 | 0.327 | 0.322 |
| **Aortic insufficiency** | | | | | | |
| Single vs Two | 5 | 1.45 (0.47, 4.46) | 1.29 (0.39, 4.26) | 0.0 | 0.430 | NA |
| Single vs Three | 3 | 9.43 (1.61, 55.36) | 9.67 (1.67, 56.15) | 0.0 | 0.724 | NA |
| Two vs Three | 3 | 1.99 (0.24, 16.37) | 2.68 (0.14, 49.73) | 37.5 | 0.206 | NA |
| **Early mortality** | | | | | | |
| Single vs Two | 14 | 0.97 (0.43, 2.19) | 0.94 (0.37, 2.40) | 0 | 0.797 | 0.532 |
| Single vs Three | 10 | 1.34 (0.37, 4.91) | 1.11 (0.30, 4.02) | 0 | 0.769 | 0.188 |
| Two vs Three | 9 | 1.39 (0.30, 6.45) | 1.22 (0.24, 6.04) | 0 | 0.785 | 1 |
| **Late mortality** | | | | | | |
| Single vs Two | 13 | 2.22 (0.95, 5.14) | 1.77 (0.60, 5.16) | 16.2 | 0.298 | 0.677 |
| Single vs Three | 11 | 1.84 (0.55, 6.08) | 1.53 (0.47, 5.00) | 0 | 0.441 | 0.142 |
| Two vs Three | 10 | 1.47 (0.49, 4.41) | 1.26 (0.38, 4.16) | 0 | 0.633 | 0.573 |
| **Cardiopulmonary bypass time (min)** | | | | | | |
| Single vs Two | 6 | -0.69 (-0.93, -0.44) | -0.59 (-1.30, 0.12) | 78.4 | 0.001 | 1.000 |
| Single vs Three | 4 | -0.93 (-1.51, -0.35) | -0.97 (-1.66, -0.28) | 22.3 | 0.276 | 0.602 |
| Two vs Three | 4 | -0.33 (-0.85, 0.18) | -0.33 (-0.85, 0.18) | 0.0 | 0.596 | 0.497 |
| **Cross-clamping time (min)** | | | | | | |
| Single vs Two | 7 | -0.81 (-1.05, -0.57) | -0.72 (-1.14, -0.31) | 48.4 | 0.085 | 0.573 |
| Single vs Three | 5 | -1.35 (-1.90, -0.79) | -1.52 (-2.42, -0.63) | 56.4 | 0.076 | 0.174 |
| Two vs Three | 5 | -0.52 (-1.00, -0.03) | -0.52 (-1.00, -0.03) | 0.0 | 0.652 | 0.050 |
| **Post-operation gradient (mmHg)** | | | | | | |
| Single vs Two | 5 | 0.28 (-0.22, 0.78) | 0.28 (-0.22, 0.78) | 3.5 | 0.387 | 0.327 |
| Single vs Three | 4 | 0.64 (0.16, 1.12) | 0.61 (-0.33, 1.55) | 71.7 | 0.014 | 0.497 |
| Two vs Three | 3 | 0.03 (-0.51, 0.58) | 0.22 (-0.86, 1.30) | 66.5 | 0.051 | 0.117 |
| **Follow-up gradient (mmHg)** | | | | | | |
| Single vs Two | 4 | 0.49 (-0.06, 1.04) | 0.49 (-0.06, 1.04) | 0.5 | 0.389 | 1 |
| Single vs Three | 3 | 0.78 (0.26, 1.30) | 0.84 (-0.05, 1.74) | 67 | 0.048 | 0.62 |
| Two vs Three | 2 | 0.17 (-0.71, 1.05) | 0.16 (-1.59, 1.92) | 74.7 | 0.047 | NA |

Abbreviations: OR, Odds Ratio; SMD, standardized mean difference; 95% CI, 95% Confidence Interval.


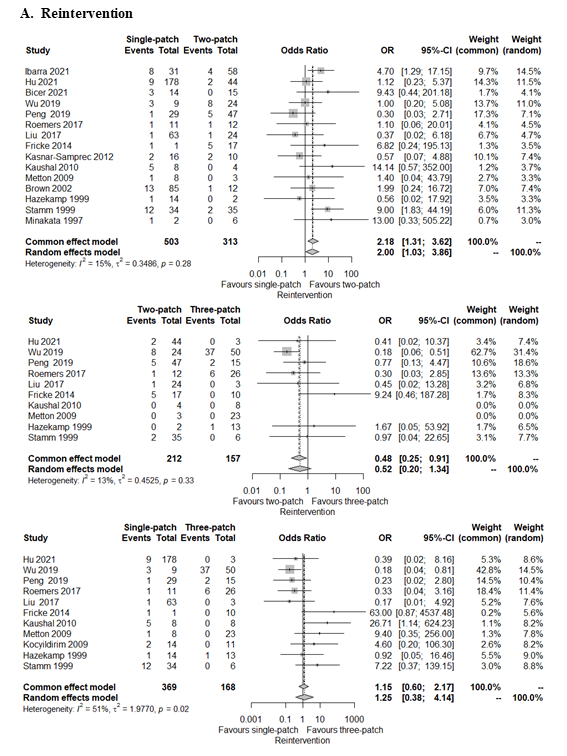


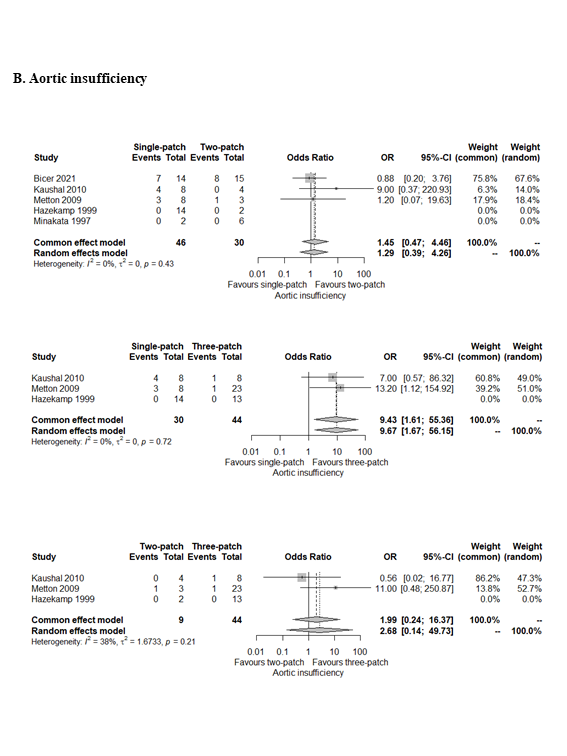


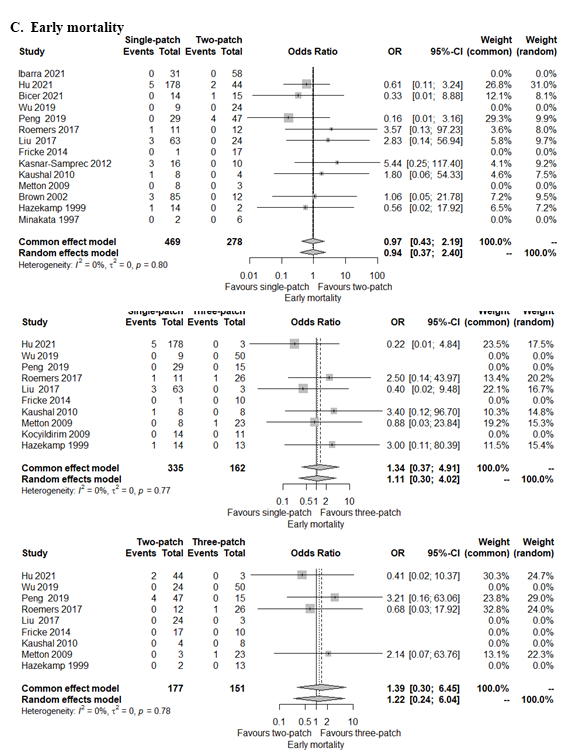


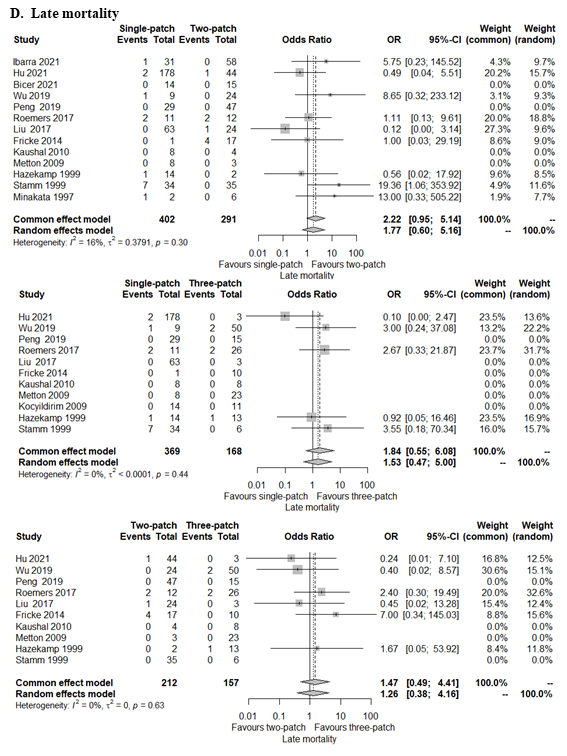


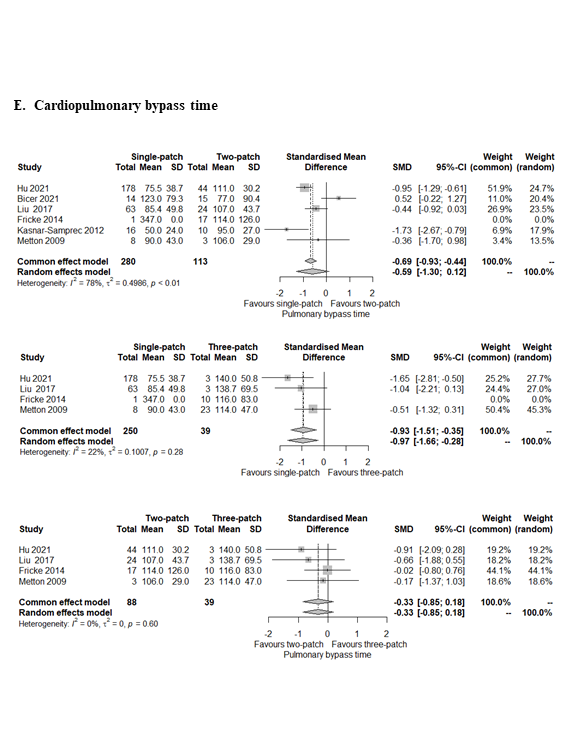


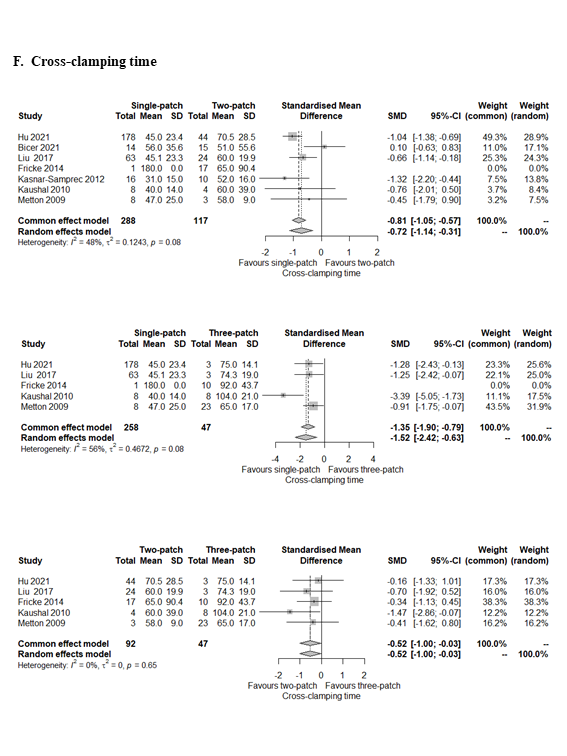


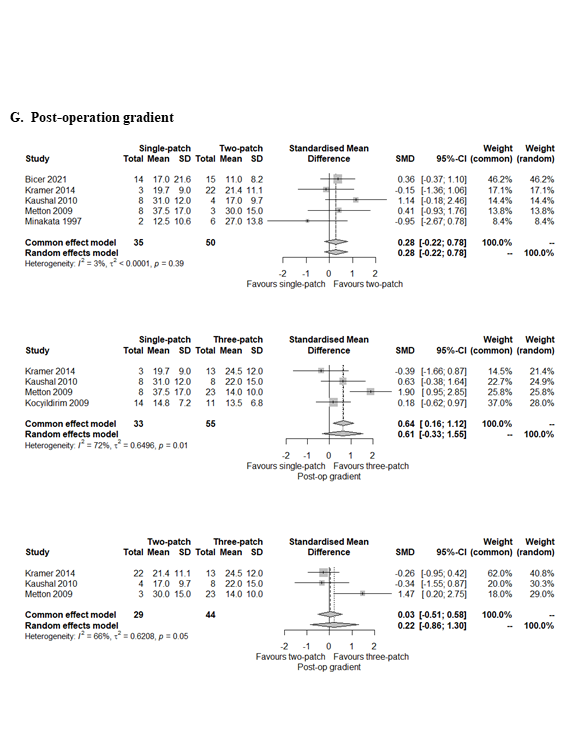


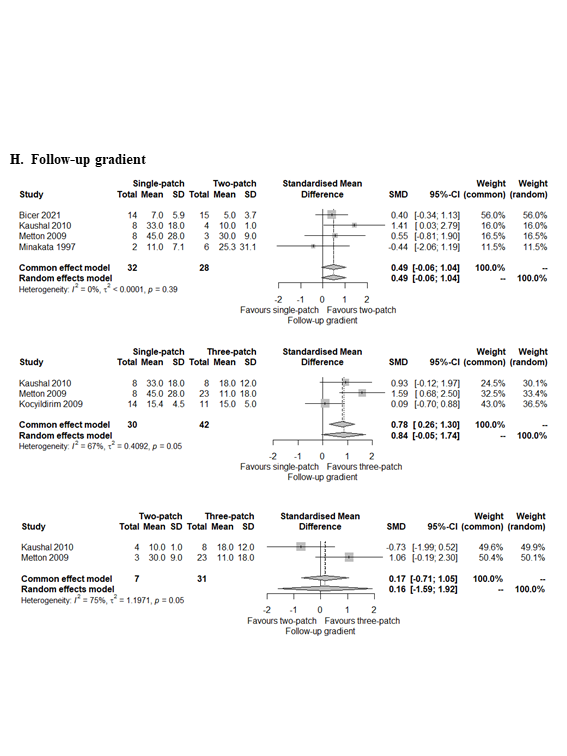


**Figure S1.** Forest plots and funnel plots for the multiple pairwise meta-analyses. (A) Reintervention, (B) Aortic insufficiency, (C) Early mortality, (D) Late mortality, (E) Cardiopulmonary bypass time, (F) Cross-clamping time, (G) Post-operation transvalvular pressure gradient and (H) Transvalvular pressure gradient at follow-up.

Abbreviations: OR, Odds Ratio; SMD, standardized mean difference; 95% CI, 95% Confidence Interval.


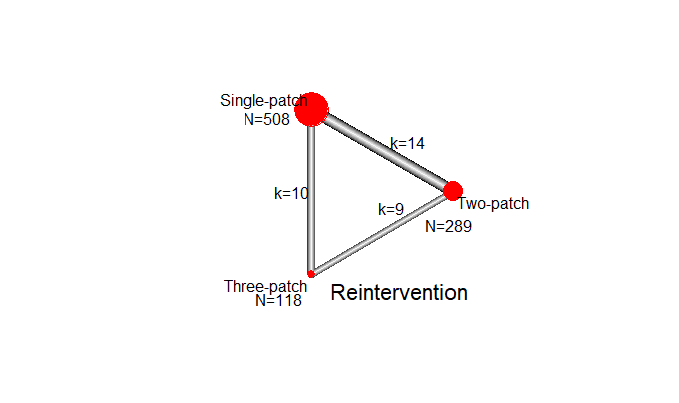

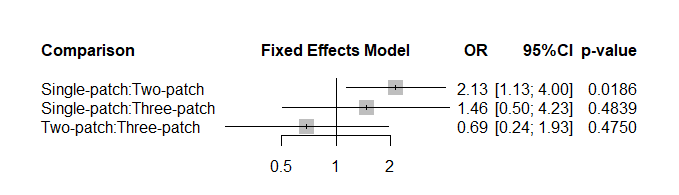


**Figure S2. Sensitive analysis omitting Wu 2019**

The width of the lines represents the number of studies comparing each pair of

treatments. The size of the red point represents the sample size in each arm

No heterogeneity was found (*τ^2^* = 0, *I^2^* = 1.3%, Q statistic =22.28 p = 0.443)

Abbreviations: OR, Odds Ratio; 95% CI, 95% Confidence Interval
